# Short-term changes in des-acyl ghrelin following bariatric surgery

**DOI:** 10.1530/EC-25-0325

**Published:** 2026-01-02

**Authors:** Guna Bīlande, Maksims Mukāns, Igors Troickis, Oļegs Kozlovskis, Egons Liepiņš, Juris Žarinovs, Valdis Pīrāgs

**Affiliations:** ^1^ Jūrmala Hospital, Jūrmala, Latvia; ^2^ Aiwa Clinic, Riga, Latvia; ^3^ University of Latvia, Faculty of Medicine and Life Sciences, Riga, Latvia; ^4^ Turība University, Faculty of Health Care, Riga, Latvia; ^5^ Riga Stradiņš University, Statistics Unit, Riga, Latvia; ^6^ Sigulda Hospital, Sigulda, Latvia; ^7^ Pauls Stradiņš Clinical University Hospital, Department of Endocrinology, Riga, Latvia

**Keywords:** des-acyl ghrelin (DAG), unacylated ghrelin, bariatric surgery, Roux-en-Y-gastric bypass (RYGB), sleeve gastrectomy (SG)

## Abstract

**Background:**

Bariatric surgery affects multiple physiological systems, including the regulation of des-acyl ghrelin (DAG). DAG has been negatively associated with excess adiposity and insulin resistance.

**Objectives:**

To assess the relationship between serum DAG concentrations and short-term weight loss following bariatric surgery.

**Setting:**

Prospective multicenter cohort study across three bariatric surgery centers in Latvia.

**Methods:**

Fasting blood samples for DAG measurement were collected 1 day preoperatively, 2 days postoperatively, and at 3 months post-surgery. Anthropometric and laboratory assessments were performed at the same time points.

**Results:**

A total of 62 patients were included; 64.5% (*n* = 40) underwent Roux-en-Y gastric bypass (RYGB), and 35.5% (*n* = 22) sleeve gastrectomy (SG). The cohort was predominantly female (67.7%). Median baseline weight and BMI were 129 kg (IQR 106–150) and 45.1 kg/m^2^, respectively. Median excess weight loss at 3 months was 40.2% (IQR 32.2–54.3%). DAG concentrations showed significant inverse correlations with preoperative weight (*r* = −0.371), BMI (*r* = −0.311), and excess weight (*r* = −0.355; all *P* < 0.05). These associations persisted across all sampling points in the RYGB group, whereas in SG patients, they were largely confined to postoperative day 2. No significant relationships were observed between DAG and relative weight-loss metrics. DAG levels were higher in females; age showed no association with DAG changes.

**Conclusion:**

DAG levels are inversely associated with absolute measures of adiposity, particularly among RYGB patients. These findings support DAG’s potential relevance as a marker of total fat burden and early postoperative metabolic response.

## Introduction

Obesity represents a major global healthcare burden associated with significant socioeconomic costs. The increasingly ‘obesogenic’ environment – characterized by excessive caloric intake and sedentary behavior – has driven a rising prevalence of severe and super obesity (BMI > 50 and >60 kg/m^2^, respectively) (1, 2, 3, 4). Obesity is a multifactorial condition underpinned by dysregulated neuroendocrine signaling. Among these mechanisms, gastrointestinal hormones play a critical role in regulating appetite and energy homeostasis (5).

For patients with severe obesity, bariatric surgery remains the most effective and durable intervention, leading to substantial weight loss, remission of metabolic comorbidities, improved quality of life, and reduced mortality (3, 6, 7, 8, 9, 10, 11, 12).

While initially bariatric surgery was considered to only alter the anatomy of the stomach and to be primarily restrictive or malabsorptive in nature, for the last decades it is now recognized to induce profound hormonal and metabolic changes beyond anatomical modification (13). The mechanisms underlying the beneficial outcomes of bariatric surgery in humans are still being uncovered (14, 15, 16, 17), but it clearly goes beyond caloric restriction and malabsorption.

One such change involves alterations in the ghrelin axis, including des-acyl ghrelin (DAG), the unacylated form of ghrelin, which was once thought to be an inactive by-product of acylated ghrelin but is now recognized as a biologically active peptide (18, 19, 20, 21). DAG, which constitutes the predominant circulating ghrelin isoform (20, 21, 22, 23), exerts insulin-sensitizing and potentially anorexigenic effects, with plasma levels inversely related to adiposity (24, 25). Reduced DAG concentrations have been implicated in obesity-associated insulin resistance (24).

Previous research has primarily focused on acylated or total ghrelin, leaving DAG dynamics insufficiently characterized, particularly in the early postoperative period following different bariatric procedures. Understanding how DAG responds to rapid weight reduction and metabolic adaptation after surgery could provide insight into the hormonal mechanisms underlying surgical efficacy.

Predicting the efficacy and outcomes of bariatric surgery remains a complex challenge. While patient motivation is a well-established determinant of success (26), identification of reliable biomarkers predictive of bariatric surgery outcomes could significantly enhance patient selection, optimize resource allocation, and improve overall surgical efficacy.

The present study aimed to investigate short-term changes in serum DAG concentrations following Roux-en-Y gastric bypass (RYGB) and sleeve gastrectomy (SG) and to evaluate their relationship with early postoperative weight loss and adiposity measures. Unlike previous studies focusing solely on baseline associations, this study explores the temporal behavior of DAG in the immediate and early postoperative period to assess its potential as a biomarker of adiposity and metabolic response after bariatric surgery.

## Materials and methods

### Study design

**Figure 1:**
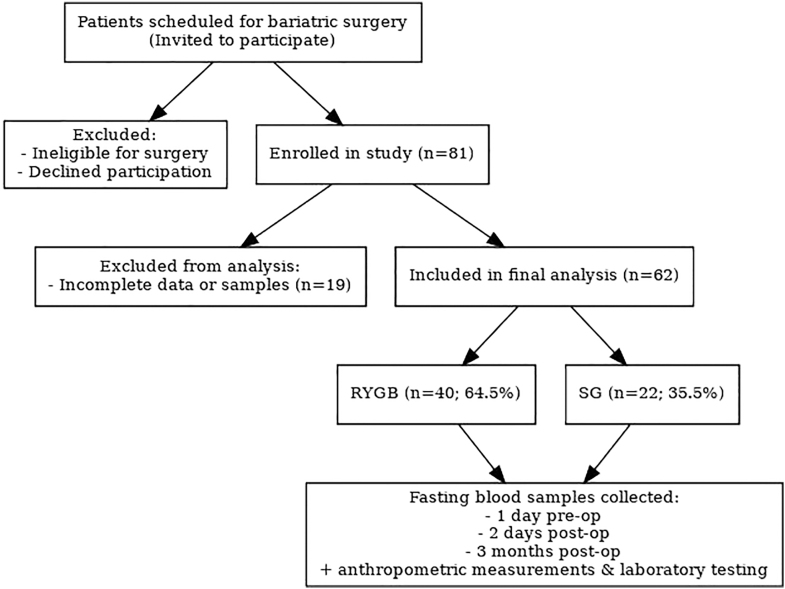
Study flow diagram.

A prospective multicenter cohort study was conducted across three bariatric surgery centers in Latvia. All patients scheduled for bariatric surgery at these institutions were invited to participate on a voluntary basis. Inclusion criteria were standardized across centers in accordance with the guidelines of the International Federation for the Surgery of Obesity (27) and included documented failure to achieve adequate weight loss despite at least 6 months of comprehensive nonsurgical management. Participants were required to be suitable candidates for anesthesia and surgery and committed to long-term health monitoring. Surgical procedures, RYGB or SG, were selected based on individual patient characteristics (comorbidities or prior abdominal surgeries) and shared decision-making with the surgeon. Patients ineligible for surgery or declining participation were excluded (Fig. 1).

Based on the previous literature, RYGB was favored over SG in patients with a history of type 2 diabetes mellitus (T2DM) or gastroesophageal reflux disease (GERD). T2DM was diagnosed according to standard criteria, including any of the following: HbA1c ≥ 6.5%, fasting plasma glucose ≥7.0 mmol/L, or a 2 h oral glucose tolerance test result ≥200 mg/dL. Patients under 18 years of age and those with a history of excessive alcohol consumption (>20 g/day) were excluded from the study (28).

Surgeons adhered to international guidelines to standardize surgical procedures and ensure comparability of outcomes. In SG, the distance from the antrum was maintained at 5 cm, while in RYGB, the biliopancreatic limb and Roux limb lengths were 100 and 150 cm, respectively.

Informed consent was obtained from all study participants. A multivitamin supplement was recommended for all patients, regardless of surgical type, with additional calcium supplementation prescribed post-RYGB. Further supplementation was individualized based on follow-up blood test results. Patients using antidiabetic or antihypertensive medications were advised to consult their primary care physician for potential dose adjustments or discontinuation post-surgery.

Fasting blood samples for DAG analysis were collected 1 day preoperatively, 2 days postoperatively, and at the 3-month postoperative at patient follow-up visit. The first two blood draws occurred during hospitalization, while the third was obtained during the outpatient surgical follow-up visit. Concurrent anthropometric measurements and other blood tests were also taken. Data collection spanned an 18-month period from March 1, 2019 to August 24, 2020 across the three participating clinics.

All clinical data, including demographic information, medical history, and routine laboratory results, were securely stored in a pseudonymized database.

The study protocol adhered to the ethical principles outlined in the 1964 Helsinki Declaration and its subsequent amendments, as approved by the Ethics Committee of the University of Latvia Institute of Cardiology and Regenerative Medicine.

### Laboratory tests

#### Blood sample collection and processing for DAG assay

Fasting blood samples were collected in tubes containing protease inhibitors (BD P800, Becton, Dickinson and Company, USA) to prevent DAG degradation. The tubes were processed according to the manufacturer’s instructions. Plasma was separated into 3–4 aliquots and stored at −80°C for subsequent DAG measurements.

#### DAG assay

DAG levels were quantified using a commercial enzyme-linked immunosorbent assay (ELISA) kit (Cat. no. RA194063500R; BioVendor – Laboratorni medicina a.s., Czechia) at the University of Latvia laboratories. Assays were performed following the manufacturer’s instructions, with samples diluted fivefold. Absorbance was measured using an ASYS Expert Plus microplate reader (Biochrom, UK). Assay precision was evaluated using quality control samples at five concentration levels following a 1:5 plasma dilution, with six replicates per run across nine independent runs. Intra-assay coefficients of variation ranged from 4.2% (ULOQ) to 6.1% (LQC), while inter-assay CVs ranged from 12.5% (HQC) to 19.4% (LLOQ).

### Statistical analysis

Data were analyzed using IBM SPSS Statistics, version 23.0 (USA). The Shapiro–Wilk test was employed to assess the normality of interval data. Given the non-normal distribution of the data and small sample size, nonparametric statistical methods were applied. Descriptive statistics included median (Me) and interquartile range (IQR). Baseline patient characteristics were compared between RYGB and SG groups using the Mann–Whitney *U* test. Specifically, age, weight, and body mass index (BMI) were analyzed. In addition, differences in DAG levels were assessed between the RYGB and SG cohorts. The Wilcoxon signed-rank test was employed to assess changes in weight, excess weight, BMI, and DAG levels over time within each group. Spearman rank correlation analysis was performed to assess the relationship between DAG levels, glucose levels, and weight loss. The chi-square test or Fisher exact test or was applied to find significant association in between type of surgery and baseline preoperative data (obesity degree, comorbidities, and sex). Correlation coefficients were interpreted as follows: |*r*| ≤ 0.30, weak; 0.31 ≤ |*r*| ≤ 0.65, moderate; |*r*| > 0.65, strong. Statistical significance was defined as *P* < 0.05. Data visualization was performed using Microsoft Office Excel 365.

## Results

### Patient characteristics

Of the 81 patients enrolled in this study, 62 patients with complete demographic and clinical data, as well as serial fasting blood samples, were included in the subsequent data analysis. Of these participants, 64.5% (*n* = 40) underwent RYGB, and 35.5% (*n* = 22) underwent SG (Table 1).

**Table 1:** Patient characteristics.

	Total		RYGB		SG		*P* value
	Me/*n*	IQR/%	Me/*n*	IQR/%	Me/*n*	IQR/%	
Patient number	62	100%	40	64.5%	22	35.5%	---
Age, years	44.6	9.9	44.5	10.2	44.9	9.6	0.837
Body weight, kg	129	106–150	134	113–155	119	105–133	0.040
Female	42	67.7%	24	60%	18	81.8%	0.079
Male	20	32.3%	16	40%	4	18.2%	
BMI, kg/m^2^	45.1	39–48	45.6	40–48	39.7	38–48	0.059
Obesity I	5	8.1%	2	5%	3	13.6%	0.036
Obesity II	15	24.2%	6	15%	9	40.9%	
Obesity III	30	48.4%	24	60%	6	27.3%	
Superobese	12	19.4%	8	20%	4	18.2%	

Preoperative median body weight and BMI were 129 kg (IQR: 106–150 kg) and 45.1 kg/m^2^, respectively. While the SG group exhibited a lower median BMI (39.7 kg/m^2^) compared to the RYGB group (45.6 kg/m^2^), this difference did not reach statistical significance (*P* = 0.059). However, patients undergoing RYGB had significantly higher preoperative body weight compared to those undergoing SG (median: 134 vs 119 kg, *P* = 0.04).

**Figure 2:**
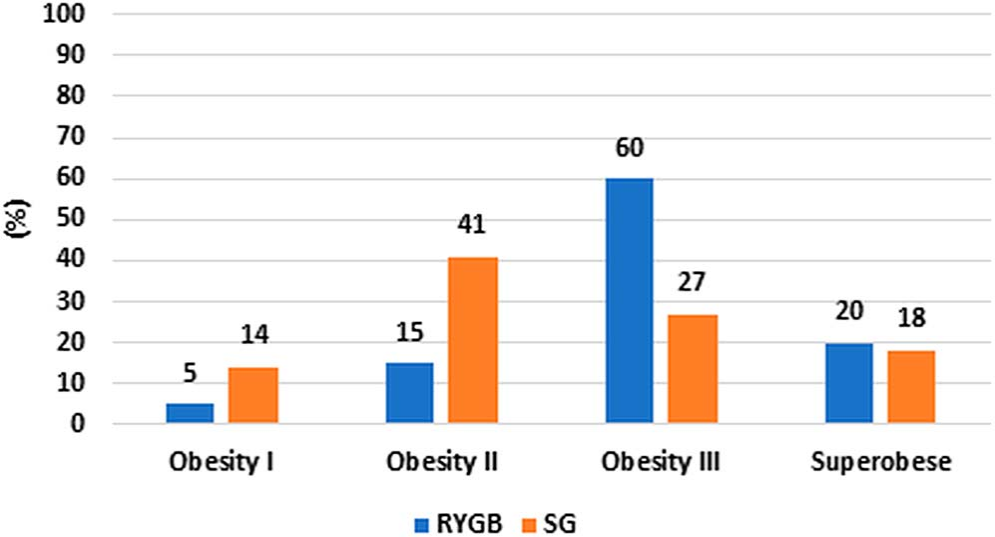
Type of surgery and BMI grade before bariatric surgery.

The distribution of patients across BMI categories according to surgical type is presented in Fig. 2. Sixty percent of RYGB surgeries were performed on patients with grade III obesity, while 41% of SG patients were classified as having grade II obesity preoperatively.

A high prevalence of comorbidities was observed (Table 2), with 98.4% (61 out of 62) of patients reporting at least one condition. The most common comorbidities included gastrointestinal (90%), cardiovascular (51.6%), musculoskeletal (48.4%), and respiratory (42%) disorders. In addition, endocrine disorders affected 23% of patients, with T2DM being the primary endocrine condition (*n* = 9), including six in the RYGB group and three in the SG group. Polycystic ovary syndrome was reported by three patients from the RYGB group. Psychiatric conditions were reported by 4.8% of patients.

**Table 2:** Comorbidities before bariatric surgery.

	All patients	RYGB	SG	*P* value
Comorbidities	98.4%	100%	95.5%	0.355
GI diseases	90.3%	97.5%	77.3%	0.018
Cardiovascular disease	51.6%	50.0%	54.5%	0.795
Endocrine disease	22.6%	27.5%	13.6%	0.342
including T2DM	14.5%	15.0%	13.6%	0.884
Respiratory disease	41.9%	32.5%	59.1%	0.042
including sleep apnea	29.0%	22.5%	40.9%	0.127
Psychiatric disease	4.8%	5.0%	4.5%	>0.999
Musculoskeletal problems	48.4%	37.5%	68.2%	0.021
Arterial hypertension	41.9%	37.5%	50%	0.34

Among gastrointestinal (GI) diseases, GERD was the most common, affecting 58.1% of patients overall (62.5% in the RYGB group and 50.0% in the SG group; *P* = 0.34). Gastritis was another prevalent GI condition (overall 40.3%). A significantly higher proportion of RYGB patients experienced GI disease compared to SG patients (97.5 vs 77.3%, respectively; *P* = 0.018).

The median percentage of excess weight loss (%EWL) at 3 months post-surgery was 40.2% (IQR: 32.2–54.3%) for the entire cohort. At the 3-month follow-up, the median weight was 110 kg (IQR: 91–128 kg) in the RYGB group and 94 kg (IQR: 83–108 kg) in the SG group. Correspondingly, the median reduction in BMI was 8 kg/m^2^ for RYGB patients and 6 kg/m^2^ for SG patients (Table 3).

**Table 3:** Weight loss for all patients and between RYGB and SG groups.

Parameter	All (me (IQR))	RYGB (me (IQR))	SG (me (IQR))	*P* value
Weight before surgery, kg	129 (106–150)	134 (114–155)	112 (105–133)	0.04
Weight 3 months after surgery, kg	104 (88–124)	110 (91–128)	94 (83–108)	0.037
TWL%	17.3 (15.5–19.3)	17.3 (15.1–19.3)	17.4 (16.2–19.3)	0.918
BMI before, kg/m^2^	45 (39–48)	46 (40–48)	40 (37–48)	0.059
BMI after 3 months, kg/m^2^	37 (32–41)	38 (34–41)	34 (29–38)	0.05
Changes in BMI, %	17.3 (15.5–19.3)	17.3 ((15.1–19.3)	17.4 (16.2–19.3)	0.918
EW before, kg	57 (38–73)	63 (39–74)	42 (33–63)	0.063
EW 3 months after, kg	33 (17–48)	38 (23–49)	24 (12–33)	0.045
EWL%	40.2 (32.2–54.3)	37.5 (32.2–51.4)	44.5 (34.1–56.9)	0.102

A median BMI reduction of 17.3% was observed across the entire cohort, with no significant sex-based differences (17.3% in females vs 17.6% in males).

Patients with lower initial weights demonstrated proportionally greater %EWL within the first 3 months post-surgery, particularly following SG. Only two of the initially superobese patients retained their preoperative obesity classification. All patients who were initially classified as having grade I obesity achieved overweight status postoperatively.

Within the first 3 months post-RYGB, all superobese patients (*n* = 12, 19.4% of the cohort) achieved substantial weight loss, with 87.5% experiencing a two-grade reduction in obesity classification (from superobese to grade II obesity). A similar pattern was observed in five patients from the grade III obesity group. In contrast, only one patient from each of the superobese and grade I obesity groups achieved a two-grade reduction following SG.

**Figure 3:**
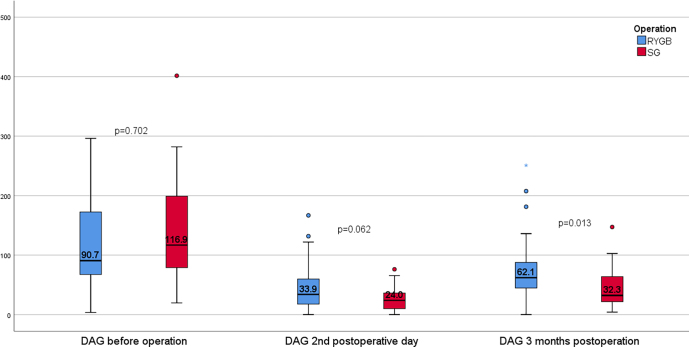
DAG changes after SG and RYGB at each sampling, pg/mL.

Analysis of DAG data revealed significant dynamic changes over time (Fig. 3), characterized by an initial precipitous decline 2 days post-surgery, followed by a gradual increase throughout the 3-month follow-up period. Despite this increase, DAG levels remained below preoperative values. A statistically significant difference in DAG levels (*P* = 0.013) was observed exclusively at the 3-month time point, with higher concentrations in the RYGB group compared to the SG group.

Correlations between DAG concentrations and weight loss parameters for all patients, as well as separately for the RYGB and SG groups, are summarized in Tables 4, 5, 6.

**Table 4:** Correlation of DAG changes for all patients.

All patients	DAG before surgery	DAG 2 days after surgery	DAG 3 months after surgery
Weight, kg	−0.371*	−0.368*	−0.315*
Weight after 3 months, kg	−0.340*	−0.336*	−0.291*
TWL%	0.000	−0.109	−0.076
BMI	−0.311*	−0.277*	−0.277*
BMI after 3 months	−0.265*	−0.216	−0.237
%BMIL	0.000	−0.109	−0.076
Excess weight	−0.355*	−0.356*	−0.314*
Excess weight after 3 months	−0.303*	−0.291*	−0.276*
EWL%	0.160	0.083	0.142

* Statistically significant correlation (*P* < 0.05).

**Table 5:** Correlation of DAG changes for RYGB patients.

RYGB	DAG before operation	DAG 2nd postoperative day	DAG 3 months post operation
Weight, kg	−0.401*	−0.415*	−0.404^†^
Weight after 3 months, kg	−0.405*	−0.390*	−0.391*
TWL%	0.107	0.024	0.076
BMI	−0.312	−0.263	−0.351*
BMI after 3 months	−0.295	−0.22	−0.307
%BMIL	0.107	0.024	0.076
Excess weight	−0.392*	−0.380*	−0.403*
Excess weight after 3 months	−0.353*	−0.335*	−0.368*
EWL%	0.245	0.135	0.271

* Statistically significant correlation (*P* < 0.05).

^†^ Statistically significant correlation (*P* < 0.01).

**Table 6:** Correlation of DAG changes for SG patients.

SG	DAG before operation	DAG 2nd postoperative day	DAG 3 months post operation
Weight, kg	−0.317	−0.593*	−0.402
Weight after 3 months, kg	−0.195	−0.491*	−0.322
TWL%	−0.212	−0.412	−0.4
BMI	−0.179	−0.489*	−0.302
BMI after 3 months	−0.15	−0.428*	−0.272
%BMIL	−0.212	−0.412	−0.4
Excess weight	−0.267	−0.543^†^	−0.368
Excess weight after 3 months	−0.156	−0.438*	−0.276
EWL%	−0.021	0.185	0.069

* Statistically significant correlation (*P* < 0.05).

^†^ Statistically significant correlation (*P* < 0.01).

In the overall cohort (Table 4), DAG levels demonstrated consistent, moderate, and statistically significant negative correlations with absolute measures of adiposity, including body weight, BMI, and excess weight, across all sampling points. These associations indicate that higher body mass was accompanied by lower circulating DAG levels both before and after surgery. The direction and strength of these correlations remained largely stable on the second postoperative day and at the 3-month follow-up, suggesting a persistent inverse relationship between DAG and total body mass over time.

In subgroup analyses, distinct patterns emerged between surgical types.

Among RYGB patients (Table 5), DAG levels showed consistent, moderate, and statistically significant inverse correlations with both body weight and excess weight at all three time points, confirming a stable relationship between DAG and total adiposity in this group. In contrast, BMI displayed a significant negative correlation with DAG only at the 3-month follow-up, while other time points showed the same negative trend without reaching statistical significance.

In the SG group (Table 6), the strongest negative correlations between DAG and anthropometric measures appeared on the second postoperative day, when DAG levels were inversely related to weight, BMI, and excess weight, both preoperatively and postoperatively. These correlations weakened by the 3-month follow-up, indicating a more transient response pattern compared with RYGB patients.

Relative weight loss measures – including TWL%, %BMIL, and EWL% – did not exhibit significant correlations with DAG levels at any time point. Although some of these correlations were negative in direction, they remained statistically nonsignificant across all DAG measurements in all patient groups.

Overall, these findings demonstrate that circulating DAG levels are closely and persistently linked to absolute measures of adiposity, particularly in RYGB patients, whereas SG patients display a more acute, short-term association in the immediate postoperative period.

A comparative analysis between the RYGB and SG groups revealed distinct patterns in the correlations between DAG levels and anthropometric parameters, particularly in the timing of the strongest associations. In the RYGB group, DAG concentrations demonstrated consistent, moderate, and statistically significant negative correlations with body weight and excess weight at all measured time points, preoperatively, on the second postoperative day, and at 3 months postoperatively. A significant inverse correlation with BMI was also observed at 3 months postoperatively. In contrast, the SG group exhibited a different trend: significant negative correlations between DAG levels and weight, BMI, and excess weight were observed predominantly on the second postoperative day, but not at baseline or 3 months post-surgery.

**Figure 4:**
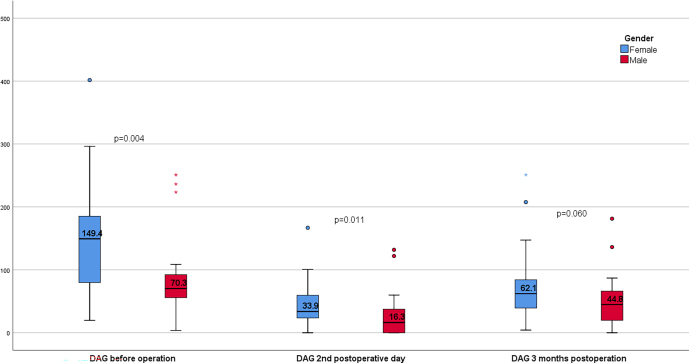
DAG changes according to patient sex.

Linear regression analysis revealed no significant association between patient age and changes in DAG levels. However, significant differences in DAG levels were observed between male and female patients. Female participants exhibited significantly higher baseline DAG levels (median = 149.4 pg/mL) compared to male participants (median = 70.3 pg/mL). This sex disparity persisted postoperatively, with lower median DAG levels in male patients at 2 days (16.3 pg/mL) and 3 months (44.8 pg/mL) compared to female patients (33.9 and 62.1 pg/mL, respectively) (Fig. 4).

A modest but statistically significant inverse correlation was observed between circulating DAG levels and fasting glucose concentrations at the first and second sampling time points in the overall patient cohort (*r* = −0.264, *P* = 0.040; *r* = −0.268, *P* = 0.037). In analyses of other research data, no correlations were observed between DAG levels, glucose levels, and weight loss.

These findings represent statistical associations between circulating DAG levels and measures of adiposity. Given the observational design of this study, the results should be interpreted as correlational rather than causal. The present analysis does not allow inference on the underlying mechanisms linking DAG dynamics to postoperative weight changes.

## Discussion

This study demonstrated a consistent inverse association between circulating DAG levels and absolute measures of adiposity – including body weight, BMI, and excess weight – in the overall cohort and among RYGB patients, whereas in SG patients, these correlations were limited to the early postoperative period, suggesting a more transient metabolic response.

The absence of a significant preoperative association between DAG levels and adiposity in the SG group may be attributed to the smaller sample size and higher interindividual variability in baseline DAG secretion, which could have reduced statistical power. It is also possible that physiological heterogeneity among SG candidates – such as differences in metabolic status or fasting insulin sensitivity – contributed to the lack of a clear baseline relationship.

While total weight loss percentage and other relative metrics did not significantly correlate with DAG levels, absolute values of body mass parameters were more strongly and consistently associated with DAG dynamics, particularly in the overall cohort and the RYGB group. These findings support the potential role of DAG as a biomarker of total adiposity rather than of percentage-based weight reduction following bariatric surgery.

Patients undergoing RYGB exhibited significantly higher DAG concentrations at 3 months postoperatively compared to SG patients. In contrast, SG patients showed significant, but transient, negative correlations between DAG and anthropometric parameters, most prominently on the second postoperative day. This may suggest an acute metabolic adjustment in the SG group, potentially related to anatomical resection of DAG-producing gastric mucosa.

These observations align with the previous literature indicating that RYGB and SG influence ghrelin axis regulation through distinct mechanisms. RYGB, incorporating both restrictive and malabsorptive features, appears to allow partial preservation of ghrelin-producing cells, while SG involves direct resection of fundic tissue where ghrelin – and likely DAG – is predominantly synthesized. Earlier studies have similarly reported lower fasting DAG levels in SG patients postoperatively (29).

Interestingly, baseline DAG levels were significantly higher in female participants compared to males, a difference that persisted postoperatively. While Takashi *et al.* reported elevated acyl ghrelin levels in women, this sex-specific pattern in DAG has not been previously highlighted and may suggest a sex-related variation in DAG secretion or clearance requiring further investigation.

Importantly, our findings emphasize that absolute body mass, not percentage-based weight loss (e.g., %TWL, %BMIL, or %EWL), correlates with DAG changes. This suggests that DAG may serve as a more reliable indicator of total adiposity burden rather than the degree of weight reduction relative to baseline. For instance, DAG concentrations were inversely correlated with both preoperative and postoperative weights, as well as BMI, at all sampling points in the RYGB group. In the SG group, however, these associations were limited to the early postoperative period.

DAG has been implicated in promoting negative energy balance and enhancing insulin sensitivity, including suppression of hepatic glucose output and lowering circulating insulin levels (30, 31, 32, 33). Our study observed a modest but significant inverse correlation between DAG and fasting glucose at the first two sampling points, supporting its metabolic relevance. However, this correlation was not maintained at 3 months, highlighting the need for further research on DAG’s temporal metabolic effects post-surgery.

In line with previous reports, our cohort exhibited greater relative weight loss among individuals with lower baseline body weight, consistent with the mathematical nature of percentage change metrics (34). However, all patients experienced clinically meaningful absolute reductions in weight and BMI, regardless of surgical type, sex, or age.

While RYGB was more frequently selected for patients with T2DM, SG patients tended to present with lower preoperative weights, consistent with clinical guidelines and earlier research (35, 36). These patterns also reflect surgical decision-making influenced by comorbidities and anatomical considerations.

A significantly higher proportion of RYGB patients in our cohort presented with gastrointestinal (GI) disease compared with SG patients (97.5 vs 77.3%, *P* = 0.018). This finding aligns with the multicenter study by Thaher *et al.*, which demonstrated that RYGB is preferentially selected for patients with substantial upper GI pathology and offers superior GERD remission compared with SG (37). Their work also highlighted higher rates of *de novo* GERD after SG, supporting the clinical rationale for choosing RYGB in individuals with pre-existing GI disease. Our data therefore mirror established patterns in surgical selection and reinforce the relevance of GI comorbidity in determining the most appropriate bariatric procedure.

This study has several limitations. First, the short follow-up period may not capture longer-term DAG dynamics or weight loss patterns. Second, due to resource constraints, we could not assess other relevant metabolic and hormonal markers or include a nonsurgical control group. Third, the relatively small sample size – particularly within the SG subgroup – limited the statistical power to detect between-group differences, even when numerical trends appeared clinically meaningful. These between-group results should therefore be interpreted with caution. In addition, the sex imbalance may further affect generalizability, and the lack of established reference ranges for postoperative DAG values complicates interpretation of absolute concentrations.

As this study was observational and correlation-based, it cannot provide mechanistic insight or establish causality regarding the role of DAG in postoperative weight regulation. Further longitudinal and mechanistic studies with larger, balanced cohorts are warranted to confirm these findings and clarify the biological significance of DAG changes following bariatric surgery.

## Conclusions

This study demonstrates a consistent inverse association between circulating DAG levels and absolute measures of adiposity, including body weight, BMI, and excess weight, in the early period following bariatric surgery. These associations were statistically significant and most consistent among patients undergoing RYGB, persisting across all three time points. In contrast, SG patients exhibited more transient associations, primarily on the second postoperative day, possibly reflecting an acute metabolic adjustment related to anatomical differences between procedures.

Importantly, DAG levels did not correlate with relative weight loss metrics such as total weight loss percentage (TWL%), BMI loss percentage (%BMIL), or excess weight loss percentage (EWL%), suggesting that DAG may serve as a biomarker more strongly associated with total adiposity than with percentage-based reductions. Sex differences were observed, with females showing higher baseline DAG concentrations, while patient age had no apparent influence on DAG dynamics.

These findings highlight the potential utility of DAG as a biomarker of adiposity burden in the early postoperative period and underscore the need for further longitudinal studies to investigate DAG’s role in energy regulation, insulin sensitivity, and its potential utility as a postoperative biomarker.

## Declaration of interest

The authors have no commercial associations that might be a conflict of interest in relation to this article.

## Funding

This work did not receive any specific grant from any funding agency in the public, commercial, or not-for-profit sector.

## Ethics statement

The study protocol adhered to the ethical principles outlined in the 1964 Helsinki Declaration and its subsequent amendments, as approved by the Ethics Committee of the University of Latvia Institute of Cardiology and Regenerative Medicine.
